# Infection with pandemic H1N1 influenza or porcine respiratory coronavirus drives localized and individualized antibody repertoires, whereas immunization promotes repertoire convergence in pigs

**DOI:** 10.1093/discim/kyag020

**Published:** 2026-10-26

**Authors:** Emily J Briggs, Shafiyeel Chowdhury, Bharti Mittal, Valentine Chaillot, Charlotte May, Kevan Hanson, Ehsan Sedaghat-Rostami, Sanis Wongborphid, Erica Bickerton, Basudev Paudyal, Christine S Rollier, Cornelis A M de Haan, Kristien Van Reeth, Sarah Keep, Matthew D J Dicks, Danish Munir, Marie Bonnet-Di Placido, Elma Tchilian

**Affiliations:** The Pirbright Institute, Pirbright, UK; Faculty of Health and Medical Sciences, School of Biosciences, University of Surrey, Guildford, UK; The Pirbright Institute, Pirbright, UK; The Pirbright Institute, Pirbright, UK; The Pirbright Institute, Pirbright, UK; The Pirbright Institute, Pirbright, UK; The Pirbright Institute, Pirbright, UK; The Pirbright Institute, Pirbright, UK; Faculty of Health and Medical Sciences, School of Biosciences, University of Surrey, Guildford, UK; The Pirbright Institute, Pirbright, UK; The Pirbright Institute, Pirbright, UK; The Pirbright Institute, Pirbright, UK; Faculty of Health and Medical Sciences, School of Biosciences, University of Surrey, Guildford, UK; Department of Biomolecular Health Sciences, Faculty of Veterinary Medicine, Utrecht University, Utrecht, The Netherlands; Department of Translational Physiology, Infectiology and Public Health, Faculty of Veterinary Medicine, Ghent University, Ghent, Belgium; The Pirbright Institute, Pirbright, UK; SpyBiotech Limited, Oxford Business Park North, Oxford, UK; The Pirbright Institute, Pirbright, UK; The Pirbright Institute, Pirbright, UK; The Pirbright Institute, Pirbright, UK

**Keywords:** influenza, coronavirus, pig, antibody repertoire

## Abstract

**Introduction:**

Respiratory viruses elicit mucosal and systemic immunity, yet how tissue compartmentalization and exposure route shape B-cell repertoires remains unclear.

**Methods:**

Using the pig model, we characterized antibody repertoires in bronchoalveolar lavage, tracheobronchial lymph nodes, spleen, and blood following intranasal infection with pandemic influenza virus (pH1N1) or porcine respiratory coronavirus (PRCV), and after intramuscular PRCV immunization.

**Results:**

Infection induced highly compartmentalized responses, with antigen-associated clones enriched in lung, lymph nodes, and spleen but with limited presentation in blood. These clones were largely private, indicating individualized responses, and displayed tissue-specific diversity. The similar tissue distributions between pH1N1 and PRCV infections suggest a conserved spatial organization, although differential lymph node involvement indicates pathogen-specific effects. In contrast, intramuscular immunization generated more convergent, public repertoires in blood, characterized by reduced clonal size diversity.

**Conclusion:**

Together, these findings show that despite stable germline usage, functional antibody responses are shaped by the route of antigen exposure, clonal selection, and host-specific factors, resulting in distinct repertoire architectures between infection and immunization.

## Graphical Abstract

**Graphical Abstract:**
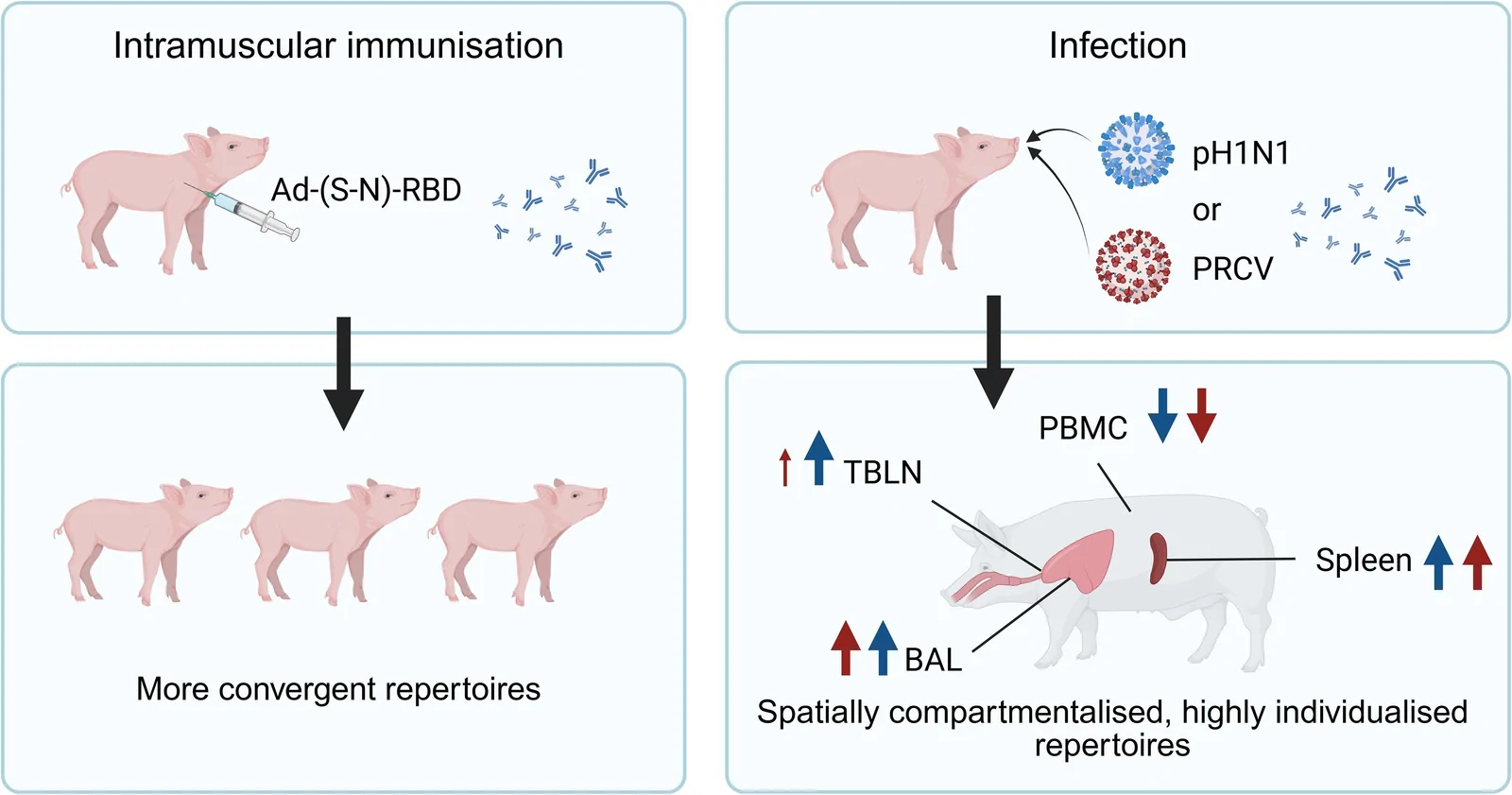

## Introduction

Respiratory viral infections remain a major global health burden, with influenza A viruses (IAV) and coronaviruses (CoV) causing periodic epidemics and pandemics. Understanding how these viruses shape the B-cell repertoire across tissues is important, as they engage both mucosal and systemic immunity. Circulating antibodies are widely used as a proxy for immune status, yet they represent only a fraction of the total response, with a substantial component residing in tissues such as the respiratory tract and its draining lymph nodes. The importance of compartmentalized immunity is illustrated by influenza immunization. Currently, two main vaccine platforms are used: intramuscularly administered inactivated influenza vaccines (IIVs) and intranasally delivered live-attenuated influenza vaccines (LAIVs). Although both provide protection, they do so through distinct immunological mechanisms. Serum haemagglutination inhibition (HAI) titres have long been considered the principal correlate of protection for IIVs, with a titre of at least 1:40 generally regarded as protective. However, HAI responses correlate poorly with protection following LAIV, which can be highly effective despite inducing minimal serum HAI seroconversion [1, 2]. This discrepancy reflects the ability of LAIV to generate local mucosal immunity, particularly nasal immunoglobulin A (IgA), without corresponding increases in serum IgA or IgG. Similarly, intramuscular severe acute respiratory syndrome CoV 2 immunization induces robust systemic antibody responses but little or no upper airway IgA [3]. These observations support the concept that the upper respiratory tract functions as a distinct immunological compartment and suggest that local immune responses [4, 5], rather than circulating antibodies alone, may provide more relevant correlates of protection for respiratory viruses [6].

Much less is known about the repertoire of tissue-resident B cells, particularly in the lung. Studies of influenza and COVID-19 have begun to address this gap, showing that lung resident memory B cells are established locally following infection and exhibit distinct transcriptional and phenotypic profiles compared with circulating populations [7]. These cells are retained within the lung and airways, do not recirculate, and can rapidly differentiate into antibody-secreting cells upon re-exposure, supporting local antibody production at sites of infection [8]. Together, these findings suggest that mucosal antibody repertoires may be distinct from those in peripheral blood, either due to local antigen-driven selection or differences in B-cell composition and maturation within tissues. However, the extent to which clonal expansion, repertoire diversity, and inter individual sharing are shaped by tissue and route of antigen exposure remains unclear. Defining the organization of compartmentalized responses, and how they differ following infection with IAV or CoV or parenteral immunization, is essential for understanding protective immunity and guiding vaccine design [9].

The domestic pig is a highly relevant large natural host animal model for studying respiratory virus infections, particularly IAV and CoV [10–13]. Pigs share many anatomical, physiological, and immunological features with humans, including similar lung structure, airway architecture, lung surface area, and distribution of airway immune cells [14–16]. They also exhibit comparable distribution of sialic acid receptors, which contributes to their natural susceptibility to IAV that are antigenically and genetically related to human strains, resulting in clinical signs, lung pathology, and immune responses similar to those observed in humans [13, 17, 18]. Pigs are natural hosts for several CoV, including porcine respiratory CoV (PRCV), which induces lung pathology comparable to that in patients with severe acute respiratory syndrome (SARS)-CoV and SARS-CoV-2 [10, 19]. These similarities, together with their role as natural hosts of IAV and CoV with zoonotic potential, make pigs an excellent translational model for understanding host–pathogen interactions and evaluating novel vaccines and therapeutics.

The Ig heavy chain locus in pigs is characterized by relatively restricted germline diversity, with a limited number of functional IGHV segments. Whilst these are closely related to the IGHV3 gene family in humans, they are referred to as IGHV1 in the pig [20]. Classical work by John E. Butler and more recent studies by Driver et al. demonstrate that only a small subset of V genes contributes to most of the antibody diversity [21–23]. Because of the small number of IGHV, IGHD, and IGHJ segments used, the combinatorial diversity in pigs is comprised of only ∼14 possibilities compared to ∼9000 in humans [22]. Consequently, repertoire diversification relies primarily on junctional diversity and somatic hypermutation rather than extensive germline gene usage. These features make the pig a useful comparative model for dissecting how antigen-driven selection and tissue specific factors shape antibody repertoire architecture in the context of limited germline variation.

In this study, using B-cell sequencing in bronchoalveolar lavage, tracheobronchial lymph nodes, spleen, and peripheral blood, we investigated the antibody repertoires in pigs following respiratory infection with either influenza virus or PRCV or following intramuscular PRCV immunization. By integrating tissue distribution, inter-animal sharing, and repertoire diversity, we show that respiratory infection is associated with localized and individualized repertoire responses, whereas intramuscular immunization promotes a more convergent response across animals.

## Results

### Antibody repertoires following pH1N1 or PRCV Blg20 infection

Ten pigs each were infected with either A/swine/Gent/53/2019 (pH1N1) or PRCV/swine/Belgium/PS-071/2020 (PRCV Blg20). At 5 and 12 days post-infection, five animals per infection group and two control uninfected animals were culled [12]. IgG and IgA sequences from bronchoalveolar lavage (BAL), tracheobronchial lymph node (TBLN), peripheral blood mononuclear cells (PBMC) and spleen samples from animals culled 5 and 12 days post-infection were sequenced for antibody repertoire analysis (Supplementary Figure S1a). Samples underwent quality control (Supplementary Figure S1b, c) and reads were normalized to enable equal representation of clonotypes across samples with different sequencing depths.

Clusters were defined as groups of heavy chain sequences sharing at least 94% identity across the full variable region, representing closely related antibody sequences. Clusters were classified as ‘selected’ (potentially antigen-specific) or ‘irrelevant’ (unlikely to be antigen-specific) based on their kinetics: clusters were classified as irrelevant if at least 10% of normalized reads were derived from pre-bleed samples or uninfected control animals. All other clusters were classified as selected (Supplementary Figure S1d–g). These selected clusters should therefore be interpreted as kinetically enriched, response-associated repertoire features rather than as definitively antigen-specific clones.

### Tissue distribution, publicity, and diversity of clusters following pH1N1 infection

Following pH1N1 infection, irrelevant clusters contained 2.1-fold more sequences from PBMC (51.1%) compared to selected clusters (24.3%) (Fig. 1a). In contrast, selected clusters were enriched in BAL, TBLN or spleen between 1.8- and 2.4-fold compared to irrelevant clusters (*Χ*^2^ = 14 912, *df* = 4, *P* < 0.0001) (**Fig. 1**b, Supplementary Table S1). Clusters containing sequences from two or more tissues were defined as shared clusters. The overall proportion of shared clusters was similar between groups (26% of irrelevant and 27% of selected clusters), although distributed differently between tissues. These results showed that clusters associated with response to pH1N1 infection were preferentially localized to BAL, TBLN, and spleen rather than the peripheral blood.

**Figure 1:**
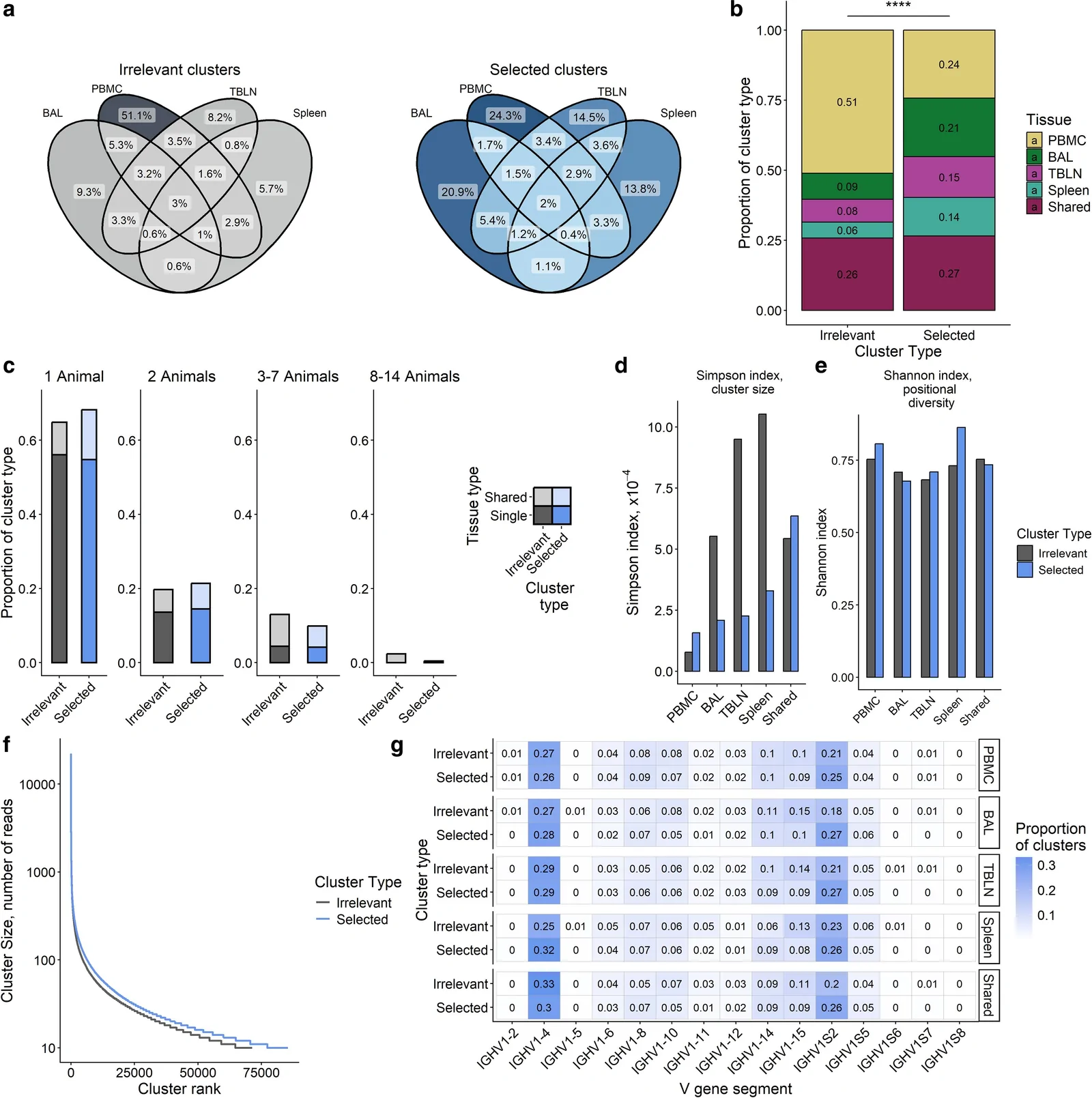
Antibody repertoire following infection with pH1N1. Selected and irrelevant clusters were analysed for their tissue distribution, sharing between animals, diversity, size, and V gene segment usage following pH1N1 infection. **A:** Distribution of irrelevant (grey) and selected (blue) clusters across tissues. Percentages of clusters of each type are indicated. **B:** Proportion of irrelevant and selected clusters containing sequences from a single tissue, or from more than one tissue (‘Shared’). Distribution across tissues was analysed by Chi-square test (*Χ*^2^ = 13206, *df* = 4, *P* < 2.2 × 10^−16^). **C:** Publicity of irrelevant and selected clusters. Clusters were grouped according to whether they contained sequences from 1 animal, 2 animals, 3–7 animals, or 8–14 animals and were further classified as restricted to a single tissue (Single) or detected across two or more tissues (Shared). **D:** Diversity of cluster size in irrelevant and selected clusters was determined by Simpson index. A lower Simpson index indicates greater diversity. **E:** Diversity of amino acid sequence in irrelevant and selected clusters was determined by Shannon diversity. **F:** Size distribution of irrelevant and selected clusters. **G:** V gene segment usage in centroid sequences of irrelevant and selected clusters that were either restricted to single tissues (PBMC, BAL, TBLN, or spleen) or shared across two or more tissues. **** indicates *P* < 0.0001.

We next assessed the extent to which clusters were shared between individuals (publicity of clusters). Overall, irrelevant clusters were more public than selected clusters, particularly among shared clusters (*Χ*^2^ = 2303.2, *df* = 3, *P* < 0.0001). Shared irrelevant clusters more frequently contained sequences from multiple animals, with 0.26% detected in 14 animals. By contrast, only 0.03% of selected clusters were detected across multiple tissues and all 14 animals (*X*^2^ = 162.98, *df* = 1, *P* < 0.001). Selected clusters were more often private or shared between only two animals, particularly amongst clusters detected in only one tissue (*Χ*^2^ = 40.143, *df* = 3, *P* < 0.0001) (Fig. 1c, Supplementary Tables S2 and S3). This suggests that multi-tissue irrelevant clusters may reflect widely shared background repertoire, whereas multi-tissue dissemination of selected clusters was largely animal-specific, indicating individualized responses to pH1N1 infection.

Cluster size diversity was quantified using the Simpson index. Selected clusters restricted to BAL, TBLN, or spleen showed greater diversity in cluster size than irrelevant clusters, indicated by a decreased Simpson index (Fig. 1d). PBMC-only and shared selected clusters showed reduced size diversity compared to irrelevant clusters (Fig. 1d). Positional diversity was analysed using the centroid amino acid sequence of each cluster, defined as the heavy chain sequence with the greatest connectivity to other sequences within that cluster. Positional diversity of selected and irrelevant clusters was assessed using the Shannon index. Selected clusters in the PBMC and spleen exhibited increased positional diversity compared to irrelevant clusters, with a modest increase also observed in TBLN. A modest decrease in positional diversity was observed in selected clusters restricted to the BAL and those shared across tissues (Fig. 1e). Furthermore, selected clusters were generally larger than irrelevant clusters (Fig. 1f). Together, these data indicate that the pattern of diversity in selected clusters is not uniform across tissues, instead showing tissue-specific differences in both cluster size distribution and amino acid sequence diversity.

V gene segment usage was analysed separately in selected and irrelevant clusters that were either restricted to a single tissue or shared across multiple tissues. Two V genes, IGHV1-4 and IGHV1S2, comprised approximately 50% of both selected and irrelevant clusters across all tissues. The next most dominant V gene segments were IGHV1-15, IGHV1-14, and IGHV1-8. Together, these five gene segments comprised approximately 75% of the repertoire (Fig. 1g). No treatment- or tissue-specific differences in V gene usage were observed, consistent with previous reports [24]. Due to the highly restricted number of D and J gene segments in the pig, only V gene segment usage was analysed.

Overall, following pH1N1 infection, antigen-associated selected clusters were enriched in BAL, TBLN and spleen, with reduced representation in peripheral blood, indicating tissue-localized responses. These clusters were more private and exhibited tissue-specific patterns in cluster size and sequence diversity, while V gene usage was dominated by a limited set of genes.

### Tissue distribution, publicity, and diversity of clusters following PRCV Blg20 infection

Cluster distribution across tissues was analysed following PRCV Blg20 infection. Irrelevant clusters were more frequently restricted to PBMC than selected clusters (1.9-fold). Clusters restricted to BAL and spleen were enriched 2.6- and 2.4-fold, respectively, in selected compared to irrelevant clusters (*Χ*^2^ = 13 206, *df* = 4, *P* < 0.0001). However, no enrichment of TBLN in selected clusters was observed (Fig. 2a). The proportion of clusters detected across multiple tissues was similar between groups (23% of irrelevant clusters and 25% of selected clusters) (Fig. 2b, Supplementary Table S4). Overall, PRCV Blg20 infection was associated with reduced representation of clusters in the PBMCs and enrichment of selected clusters in BAL and spleen, but not in TBLN.

**Figure 2:**
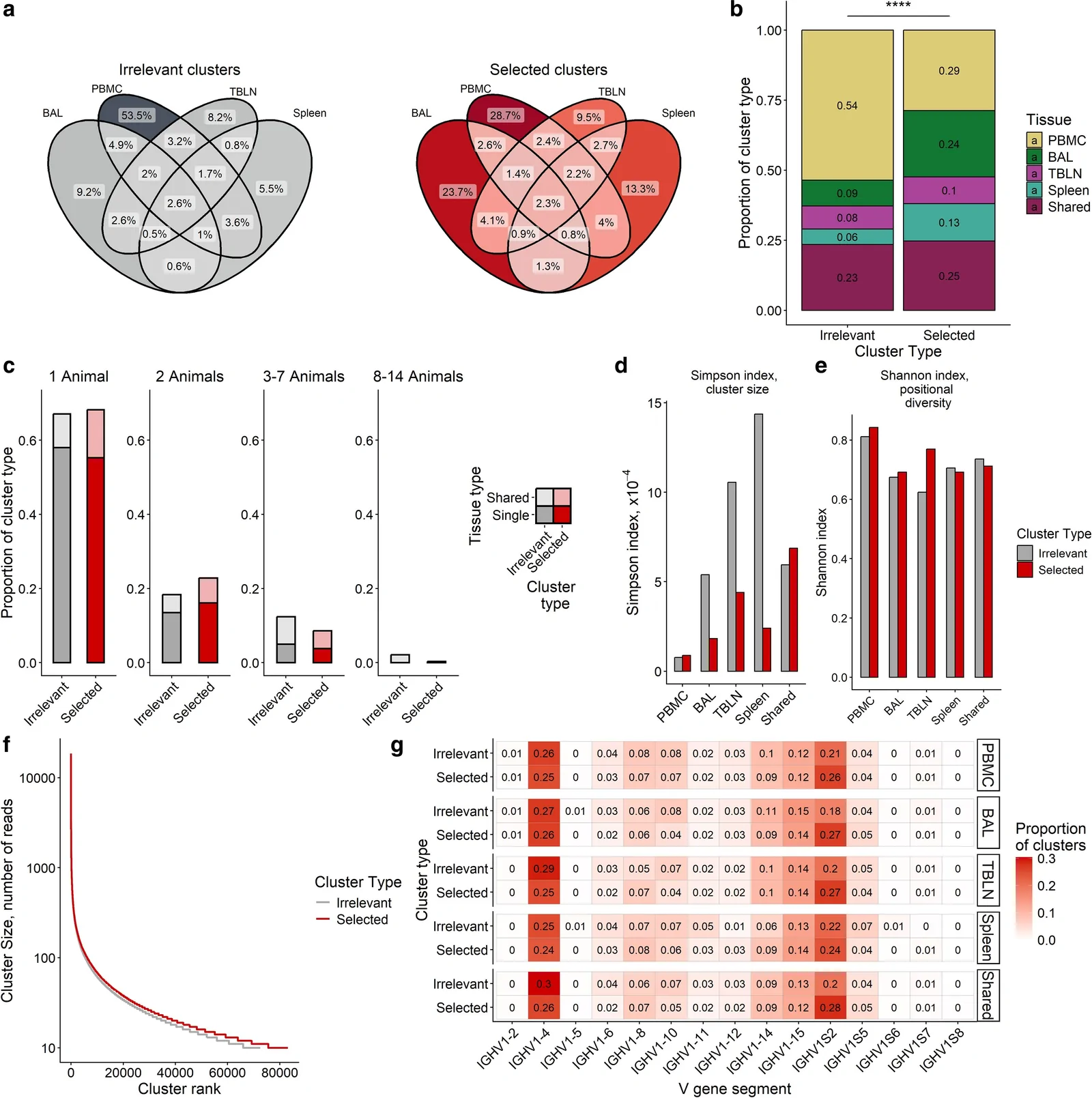
Antibody repertoire following infection with PRCV Blg20. Selected and irrelevant clusters were analysed for their tissue distribution, sharing between animals, diversity, size, and V gene segment usage following PRCV Blg20 infection. **A:** Distribution of irrelevant (grey) and selected (red) clusters across tissues. Percentages of clusters of each type are indicated. **B:** Proportion of irrelevant and selected clusters containing sequences from a single tissue, or from more than one tissue (‘Shared’). Distribution across tissues was analysed by Chi-square test (*Χ*^2^ = 13206, *df* = 4, *P* < 2.2 × 10^−16^). **C:** Publicity of irrelevant and selected clusters. Clusters were grouped according to whether they contained sequences from 1 animal, 2 animals, 3–7 animals, or 8–14 animals and were further classified as restricted to a single tissue (Single) or detected across two or more tissues (Shared). **D:** Diversity of cluster size in irrelevant and selected clusters was determined by Simpson index. A lower Simpson index indicates greater diversity. **E:** Diversity of amino acid sequence in irrelevant and selected clusters was determined by Shannon diversity. **F:** Size distribution of irrelevant and selected clusters. **G:** V gene segment usage in centroid sequences of irrelevant and selected clusters that were either restricted to single tissues (PBMC, BAL, TBLN, or spleen) or shared across two or more tissues. **** indicates *P* < 0.0001.

Distribution of irrelevant cluster publicity was analysed in the PRCV Blg20 repertoire. A small fraction of irrelevant clusters (190, 0.26%) was highly public, containing sequences from multiple tissues and all 14 animals compared to 10 in selected clusters (0.012%) (*Χ*^2^ = 188.16, *df* = 1, *P* < 0.0001). In contrast, selected clusters shared across tissues were more often restricted to a single animal than irrelevant clusters (*Χ*^2^ = 2190.8, *df* = 3, *P* < 0.0001) (Fig. 2c, Supplementary Table S5, S6).

Selected clusters restricted to BAL, TBLN, or spleen exhibited higher diversity in cluster size than irrelevant clusters, indicated by decreased Simpson index (Fig. 2d). Positional amino acid diversity was increased in selected clusters from PBMC, BAL, and TBLN, with little difference observed in spleen-only or shared clusters (Fig. 2e). Cluster size was generally larger in the selected clusters compared to irrelevant (Fig. 2f). Together, these findings indicate that selected clusters in the PRCV Blg20 repertoire were associated with increased diversity in both cluster size and amino acid sequence. This is consistent with a broader and more heterogeneous repertoire associated with PRCV Blg20 infection compared to irrelevant clusters. V gene usage in the PRCV Blg20 repertoire was dominated by five V gene segments, with IGHV1-4, IGHV1S2, IGHV1-15, IGHV1-14, and IGHV1-8 comprising approximately 75% of the repertoire. Again, no tissue- or treatment-specific differences were observed (Fig. 2g).

Together, these findings indicate that selected clusters differed consistently from irrelevant clusters following PRCV Blg20 infection. Selected clusters were detected less frequently in PBMCs and more frequently in BAL and spleen, but not TBLN. Selected clusters were largely private and showed increased clonal and sequence diversity, while overall V gene usage remained restricted.

### Comparison of antibody repertoires following pH1N1 and PRCV Blg20 infection

We next compared the repertoires following pH1N1 and PRCV Blg20 infections. Tissue distribution patterns were broadly similar between the two infections (Fig. 3a). In both datasets, selected clusters were more frequently restricted to BAL and spleen and less frequently to PBMC compared to irrelevant clusters, while the proportion of shared clusters remained largely unchanged. Selected clusters were enriched in TBLN only in the pH1N1 repertoire, but not in the PRCV Blg20.

**Figure 3:**
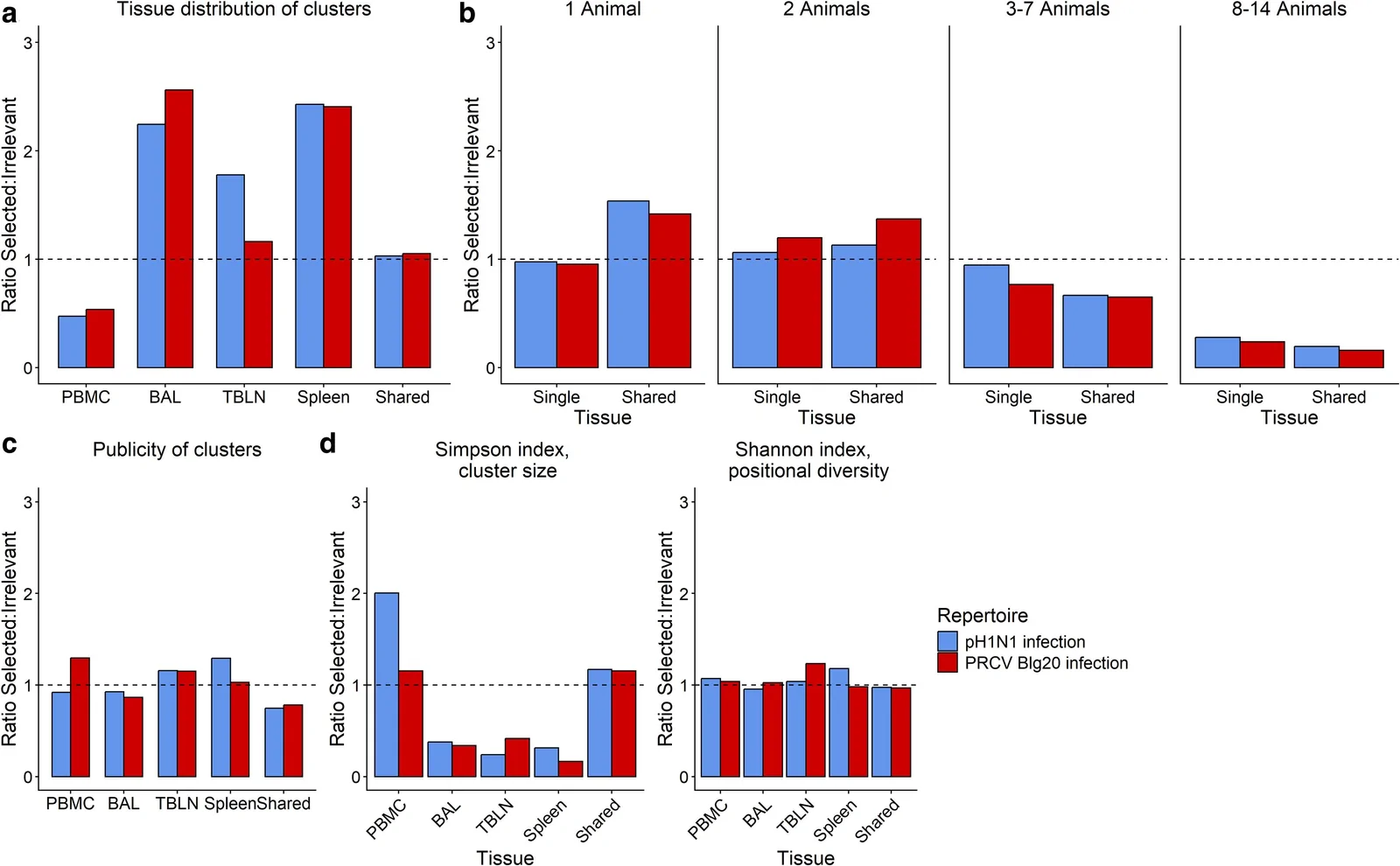
Comparison of antibody repertoires following pH1N1 or PRCV Blg20 infection. Ratios between selected and irrelevant clusters were compared across the antibody repertoires following infection with either pH1N1 or PRCV Blg20. **A:** Ratio of the proportion of selected to irrelevant clusters restricted to a single tissue or detected across more than one tissue (‘Shared’). **B:** Ratio of the proportion of public selected to public irrelevant clusters across tissue categories. **C:** Ratio of the proportion of selected to irrelevant clusters shared between two or more animals across tissue categories following infection with either pH1N1 or PRCV Blg20. **D:** Ratio of diversity between selected and irrelevant clusters, shown for cluster size (Simpson index) and centroid amino acid sequence diversity (Shannon index). A lower Simpson index indicates greater diversity. In all panels, bars above the dotted line indicate enrichment in selected clusters, whilst bars below the dotted line indicate enrichment in irrelevant clusters.

Overall publicity patterns were similar between the two infections (Fig. 3b). In both datasets, selected clusters shared across tissues were more often private than irrelevant clusters. Selected clusters were more frequently shared between two animals (indicated by ratios of selected to irrelevant clusters above the dashed line), but less frequently shared across larger numbers of animals. In both infections, selected clusters in BAL and shared clusters were less public than irrelevant clusters, whereas selected clusters in TBLN were more often public in both datasets (Fig. 3c). However, patterns of publicity also showed distinct features. In the pH1N1 repertoire, selected PBMC clusters were less public than irrelevant clusters, whereas the opposite pattern was observed following PRCV Blg20 infection. In the spleen, selected clusters were more public in pH1N1 but no different from irrelevant clusters in PRCV Blg20. This indicates that, despite common tissue-associated patterns of publicity, inter-animal sharing in blood and spleen varied according to pathogen.

Patterns of diversity in cluster size and centroid amino acid sequence were also similar between pH1N1 and PRCV Blg20 infections (Fig. 3d). Selected clusters in BAL, TBLN, and spleen were more diverse in size than the corresponding irrelevant clusters, indicated by a ratio of selected versus irrelevant clusters less than one. However, in the pH1N1 repertoire, PBMC-only clusters were less diverse than irrelevant clusters, indicated by an approximate 2-fold difference in Simpson index, which was not observed in the PRCV Blg20 repertoire. In both repertoires, selected clusters were generally larger than irrelevant clusters (Figs. 1f and 2f). Positional diversity of the centroid amino acid sequence was increased in the selected clusters in both repertoires. However, the magnitude and location of these differences varied: In the pH1N1 dataset, increased diversity was most evident in spleen-restricted clusters, whereas in the PRCV Blg20 dataset this was more pronounced in the TBLN.

V gene segment usage in both infections was dominated by IGHV1-4 and IGHV1-S2, which accounted for more than 50% of the repertoire, followed by IGHV1-15, IGHV1-8, and IGHV1-10. This pattern is consistent with previous reports, and no clear differences were observed between tissues or treatments (Figs. 1g and 2g) [22, 25–27].

In summary, selected clusters were enriched in BAL and spleen and reduced in PBMC in both infections, indicating localization of the response to lymphoid and site-specific compartments. Selected clusters were generally less public than irrelevant clusters, particularly when detected in multiple tissues, demonstrating that infection-associated responses were individual-specific. Diversity analyses further supported tissue-specific patterns of response. Despite some differences between pH1N1 and PRCV Blg20, including no TBLN enrichment in PRCV Blg20, the overall architecture of the response was similar between the two infections.

### Antibody repertoire following prime-boost immunization against PRCV

Following analysis of infection-induced responses, antibody sequences were assessed in five animals immunized twice 4 weeks apart with an adenoviral vector encoding PRCV 135 spike (S) and nucleocapsid (N) and decorated with spike receptor–binding domain (RBD) Ad-(S-N)-RBD [28] (Supplementary Figure S2a). The immunogenicity and protective efficacy of this vaccine, including the induction of high IgG and IgA antigen-specific serum antibody responses have been described previously [28]. The present study focuses on characterizing the associated antibody repertoires. PBMCs were collected at zero, seven, 14, 21, 25, 29, 36, and 39 days post-prime immunization. Spike-binding B cells were isolated by FACS from PBMC samples isolated 4 and 11 days post-booster immunization and their paired heavy and light chains sequenced for comparison with the bulk repertoires. Antibody repertoire was assessed from IgG and IgA heavy chain sequences at all time points. Following quality control of sequencing data (Supplementary Figure S2b and c), clusters from PBMC obtained post-immunization were defined as selected where less than 10% of reads were derived from pre-bleed samples, and two peaks were observed: one between 7 and 21 days after the prime dose, and a second 4 days after the boost immunization (Supplementary Figure S2d) [29]. All other clusters were defined as irrelevant (Supplementary Figure S2e). Clusters were analysed for isotype composition, publicity, and diversity.

Irrelevant clusters primarily contained sequences of a single isotype, either IgG or IgA (Fig. 4a). Selected clusters were also often restricted to one isotype, but more frequently contained both IgG and IgA sequences, with 1.7-fold more selected clusters than irrelevant containing both isotypes (*Χ*^2^ = 212.4, *df* = 9, *P* < 0.0001, Supplementary Table S7, Supplementary Figure S3), indicating representation across multiple class-switched isotypes. A higher proportion of selected clusters were public, with 1.6-fold more selected clusters than irrelevant containing sequences from two animals (*P* < 0.0001) (Fig. 4b, Supplementary Table S8). Increased publicity in selected clusters indicated that prime-boost immunization elicited a more convergent repertoire across animals, as compared with infected pigs.

**Figure 4:**
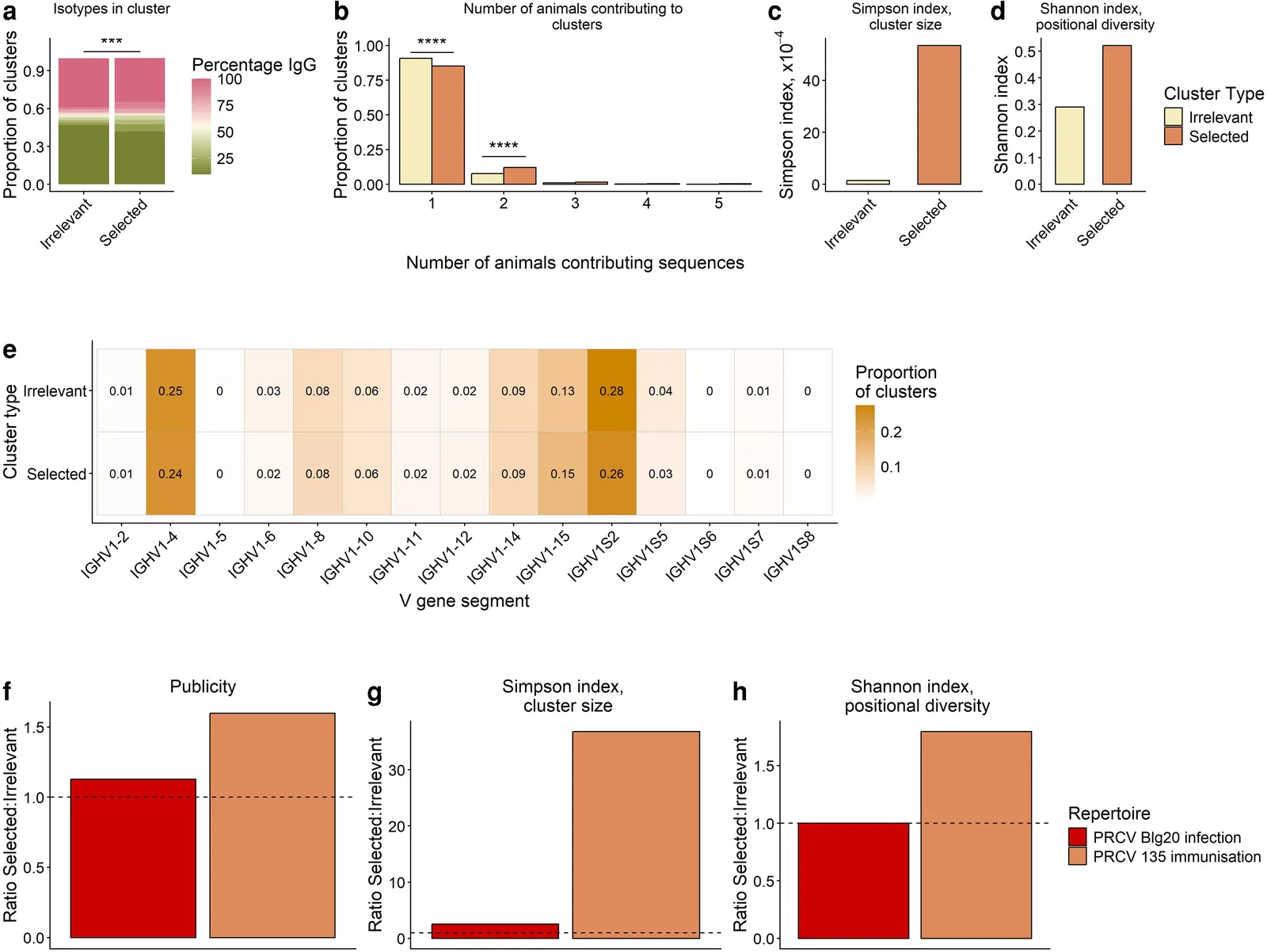
Antibody repertoire in PBMCs following prime-boost immunization against PRCV 135 or infection with PRCV Blg20. Irrelevant and selected PBMC clusters were compared following PRCV 135 immunization or PRCV Blg20 infection. **A:** Isotype usage in irrelevant and selected clusters. Isotype usage was analysed by Chi-square test (*Χ*^2^ = 212.4, *df* = 9, *P* < 2.2 × 10^-16^). **B:** Number of animals contributing sequences to each cluster. Distribution of sequences across 1–5 animals was compared by Fisher exact test for overall distribution and between cluster types for each number of animals. **C:** Cluster size diversity measured by Simpson index, where a higher value indicates lower diversity. **D:** Amino acid sequence diversity measured by Shannon index. **E:** V gene usage in centroid sequences of irrelevant and selected clusters following PRCV 135 immunization. **F:** Ratio of PBMC-only clusters containing sequences from two or more animals. **G:** Ratio of cluster size diversity between selected and irrelevant PBMC-only clusters, measured by Simpson index. **H:** Ratio of amino acid sequence diversity between irrelevant and selected PBMC-only clusters, measured by Shannon index. In panels F-H, values greater than 1 indicate higher values in selected clusters than in irrelevant clusters. **** indicates *P* < 0.0001.

Diversity in cluster size was decreased in selected compared to irrelevant clusters, indicated by increased Simpson index (Fig. 4c). In contrast, centroid amino acid sequence was more diverse in selected clusters as measured by Shannon index (Fig. 4d). Immunization was therefore associated with diversification at the sequence level. Thus, although selected clusters following immunization were more shared across animals and less diverse in size, they were more diverse in sequence composition, indicating that immunization elicited similar overall responses in different animals while maintaining sequence diversity within those responses.

V gene segment usage was analysed in selected and irrelevant clusters following immunization. As with infection, the repertoire was dominated by IGHV1-4 and IGHV1-S2, followed by IGHV1-15, IGHV1-8, and IGHV1-10.

Together these data suggest that following prime-boost intramuscular immunization, selected clusters in PBMC were more public and often contained both IgG and IgA, indicating a convergent, class switched response. These selected clusters showed reduced size diversity but increased sequence diversity, while V gene segment usage remained similar to infection.

### Comparison of PRCV infection and immunization repertoires

Repertoires following PRCV infection and immunization were compared in clusters containing sequences from PBMC. In both infection and immunization repertoires, a higher proportion of selected clusters than irrelevant clusters were shared between two or more animals (Fig. 4f). This increase was more pronounced following immunization than infection (1.6-fold and 1.1-fold, respectively). Despite this, the overall proportion of public clusters was approximately 4-fold lower in the immunization repertoire than the infection repertoire (*P* < 0.0001).

Selected clusters had lower size diversity than irrelevant clusters following immunization, indicated by an increased Simpson index (Fig. 4g). In contrast, diversity in amino acid sequence was 1.8-fold higher in selected clusters than irrelevant following immunization but not infection (Fig. 4h).

## Discussion

This study demonstrates that respiratory viral infection and intramuscular immunization generate distinct repertoire architectures in pigs, with infection producing localized and individualized responses and immunization promoting greater convergence across animals. Following pH1N1 or PRCV Blg20 infection, response-associated selected clusters were consistently enriched in BAL and spleen, with reduced representation in peripheral blood. This supports a model in which B-cell responses are primarily localized to sites of antigen exposure and secondary lymphoid organs, rather than being broadly reflected in the circulation. The similarity in tissue distribution between the two infections suggests that this spatial organization is a general feature of respiratory immune responses, although the stronger enrichment in TBLN following pH1N1 indicates pathogen-specific differences in lymphoid involvement. This might be due to the more generalized response following PRCV Blg20 infection, while pH1N1 was mainly restricted to the respiratory tract [12, 30]. Our findings are consistent with observations from influenza models where respiratory infection generated highly compartmentalized B-cell responses. Distinct and largely non overlapping clones identified in lung, spleen, and lymph nodes support the concept that antigen-driven responses are shaped locally within tissues rather than uniformly distributed across the organism [31, 32].

Publicity analysis further showed that infection-associated selected clusters were less shared across animals than irrelevant clusters. This indicates that recent antigen-driven responses are more individualized, likely reflecting differences in antigen exposure, germline repertoire, and stochastic aspects of clonal selection. The observation that irrelevant clusters were more public is consistent with the presence of common baseline clonotypes that are widely shared, as naïve repertoires across individuals contain a core of shared or ‘public’ sequences despite overall high diversity [33]. In contrast, antigen-experienced selected clusters were more private due to selection and expansion of distinct clones in each individual [34]. Despite some variation between tissues and pathogens, selected clusters were generally restricted to fewer animals, particularly when detected across multiple tissues, reinforcing the idea that functional responses are personalized. The high mutation rate of RNA viruses likely results in exposure to heterogeneous virus populations, which may contribute to the personalization of the antibody response.

In contrast to infection, prime-boost intramuscular immunization induced a more convergent PBMC repertoire across animals. Selected clusters were more frequently public, indicating that vaccination drives common responses, likely due to controlled and repeated antigen exposure. Such convergent or ‘public’ antibody responses following vaccination have been reported, with shared clonotypes identified after influenza, pseudorabies, and SARS-CoV-2 immunization [35–38]. At the same time, immunization was associated with reduced cluster size diversity but increased sequence diversity, consistent with expansion of dominant clones together with ongoing diversification, a pattern also observed following repeated antigen exposure and boosting in vaccine settings [35]. Convergence observed following immunization may partly reflect the delivery of a genetically defined antigen by the adenoviral vector, resulting in a more homogeneous antigen exposure across animals than occurs during natural infection. The presence of both IgG and IgA within selected clusters further indicates that immunization can engage multiple class-switched compartments, in agreement with studies showing that vaccination can induce both systemic and mucosal class-switched responses depending on antigen and regimen [39]. In our study, comparisons between PRCV infection and immunization were limited to PBMC only, which represent a minority of the total B-cell population. The more limited antigen exposure following immunization may partly explain the greater repertoire convergence observed compared with infection, although other factors are also likely to contribute. Indeed, convergence of the antibody repertoire following intramuscular immunization with a live attenuated vaccine has previously been reported [36]. Future work should extend this analysis to inbred swine and different tissues, including after respiratory immunization or infection with a DNA virus, to better define how route of antigen exposure shapes the repertoire across anatomical sites.

Studies of porcine antibody repertoires remain relatively limited, but several common features have emerged. In pigs, the Ig locus contains a relatively small number of functional V, D, and J genes (Supplementary Table S9), meaning that repertoire diversification depends less on combinatorial diversity and more on junctional diversity and somatic hypermutation [20, 21, 40]. Early work therefore focused mainly on spectratyping and CDRH3 length distribution, which showed repertoire perturbations after infection with porcine reproductive and respiratory syndrome virus (PRRSV), swine influenza virus or porcine circovirus 2 (PCV2), but did not provide sequence-level resolution of the responding populations [23, 41]. More recent sequencing-based studies in porcine epidemic diarrhoea virus (PEDV) vaccination or infection also showed that porcine repertoires are dominated by a limited set of IGHV genes and that changes in bulk V-gene usage after antigen exposure may remain relatively modest [25, 26]. In the recent study by Herbet *et al.* [36], kinetic clustering of heavy-chain repertoires after pseudorabies virus vaccination and challenge identified numerous public clusters shared between pigs, indicating that convergent responses can emerge in this species despite restricted germline usage. Our findings are broadly consistent with this general framework, as we also observed limited changes in overall V-gene usage. However, our data further show that major differences in the porcine antibody response can be revealed through tissue distribution, inter-animal sharing and repertoire diversity. In this context, convergence appears to be one possible feature of porcine humoral immunity, but not a universal one, as the responses observed here following respiratory infection were more strongly characterized by tissue compartmentalization and individuality, whereas immunization promoted greater sharing across animals.

V gene segment usage showed no major changes across tissues or infection type and was dominated by a limited set of IGHV1 genes, including IGHV1-4 and IGHV1-S2, IGHV1-15, IGHV1-8, and IGHV1-10. This suggests that porcine antibody responses arise from a relatively fixed germline pool, with repertoire changes driven by clonal selection rather than gene recruitment, consistent with studies in PRRSV and PEDV infections and baseline analyses [22, 24, 25, 27]. In addition, the increased diversity in amino acid sequence across the full variable region despite minimal differences in V gene usage supports the dependence on junctional diversity and somatic hypermutation as mechanisms for diversification of the porcine antibody repertoire.

Overall, these results show that respiratory infection with influenza virus or PRCV generates spatially compartmentalized, diverse, and highly individual-specific antibody repertoire responses, whereas intramuscular PRCV immunization promotes greater convergence and shared responses across animals. Tissue localization, particularly within the respiratory tract and draining lymph nodes, emerges as a key determinant of clonal expansion and diversity. Despite stable germline usage, functional antibody responses are shaped by local antigen exposure, clonal selection, and host-specific factors, resulting in distinct repertoire architectures between infection and immunization.

## Methods

### Cells, viruses, and recombinant proteins

Swine testis (ST) cells were maintained in complete ST media (Advanced MEM [Gibco] supplemented with 5% FBS, 1% penicillin/streptomycin solution, and 1% L-glutamine). Cells were passaged once per week to no more than passage 30.

Field strains Influenza A/swine/Gent/53/2019 (pH1N1) and PRCV strain PRCV/swine/Belgium/PS-071/2020 (PRCV Blg20) were provided by K. Van Reeth (Ghent University). PRCV 135 strain 86/13508 is a laboratory strain of PRCV provided by S. Cartwright (Animal and Plant Health Agency, APHA), PRCV strain ISU-1 is a laboratory strain of PRCV purchased from BEI Resources (NR-43286). pH1N1 virus was cultured in MDCK cells. PRCV viruses were cultured in ST cells using PRCV growth media (EMEM (Sigma-Aldrich) supplemented with 0.02% yeast extract, 10% tryptose-phosphate broth, 1% L-glutamine, 1% penicillin-streptomycin solution, and 1% nystatin solution). Viral titre was quantified by plaque assay in ST cells [19].

### *In vivo* infection and immunization studies in pigs

Samples from two *in vivo* studies were used for this work (Supplementary Figures S1a and S2a) [12, 28]. All *in vivo* studies were performed in accordance with the U.K. Government Animal (Scientific Procedures) Act 1986 under Project License PP7764821and PP2064443 and were approved by ethical review processes at The Pirbright Institute and Vetquest. The Pirbright Institute conforms to Animal Research: Reporting of Animal Experiments guidelines. All animals included in this work were 5–7 weeks female pigs genetically composed of one-quarter Large White, one-quarter Landrace, and one-half Hampshire breeds. All pigs were serologically negative for PRCV and pH1N1.

All animals were assessed for clinical signs including coughing, sneezing, nasal and eye discharge, rectal temperature, faecal consistency, appetite and demeanour. Endpoints based on pyrexia, behaviour, anorexia, and digestive or respiratory signs were specified in project licences PP7764821 and PP2064443. Virus was administered intranasally (1 ml per nostril) following sedation with 3 mg/kg Zoletil and 1.5 mg/kg Stresnil, and inoculum was back-titrated to confirm delivery of equivalent viral load to all animals.

Fifteen pigs were intranasally infected with 8 × 10^6^ plaque-forming units (*PFU*) each of either pH1N1 or PRCV Blg20. At 1, 5, and 12 days post-infection, five pigs per infection group and two uninfected pigs were humanely culled [12]. Samples from pigs sacrificed at 5 and 12 days post-infection were used for antibody repertoire analysis. PBMCs, BAL, TBLN, and spleen samples were collected from all animals at post-mortem, with an additional PBMC sample collected from the day 12 group at 7 days post-infection (Supplementary Figure S1a).

In the PRCV immunization study, five pigs were immunized with replication-deficient adenovirus encoding full-length spike and nucleocapsid proteins and decorated with receptor-binding domain from PRCV 135 using the DogTag/DogCatcher system, Ad-(S-N)-RBD135 [28]. All animals received a homologous booster dose at 25 days post-first dose. PBMC samples were collected at the indicated times throughout the study (0, 7, 14, 21, 25, 29, 36, and 39 days post-prime immunization) and used for antibody repertoire analysis (Supplementary Figure S2a).

### Library preparation for antibody repertoire analysis

RNA was extracted using the Qiagen RNEasy kit (Qiagen, 74104) from up to 1 × 10^7^ cryopreserved cells (from the infection study) or cells preserved in RNALater (for the immunization study) as outlined in Supplementary Figures S1 and S2. RNA was isolated according to the manufacturer’s instructions, quantified by NanoDrop, and stored at −80°C. As the infection studies were performed at SAPO4 containment, Triton X-100 was added to buffer RLT to final concentration of 1%, to facilitate movement of samples out of virus-handling laboratories.

cDNA was synthesized from 50 ng of RNA using SensiScript RT (Qiagen, 205213) supplemented with 1 μl per sample Random Primer 9 and Oligo dT(23)VN (New England Biolabs, S1254S and S1327S) and 0.25 μl per sample RNaseOUT (Invitrogen, 10777019) as described previously [42]. Samples were incubated with cDNA synthesis mix for an hour at 37°C and stored at −20°C until required.

Nested polymerase chain reaction (PCR) was performed to amplify the IgG and IgA variable domain sequences using an adapted protocol from Illumina 16S sequencing. Primers (Table 1) were designed to amplify the whole Ig heavy chain variable region with forward primers in the V leader sequence, and a portion of the constant region (Table 2, 3, Supplementary Figure S4). Overhangs facilitating addition of indexing primers were added in a second PCR (Table 4, 5), and Illumina indexing primers uniquely identifying each sample in a third PCR, as previously described [42]. Before and after the third PCR, samples were cleaned using AmpureXP beads (Beckman Coulter, A63880) at a sample:beads ratio of 1:0.65, according to manufacturer’s instructions. Presence of PCR product was confirmed before each bead clean step by agarose gel electrophoresis.

**Table 1:** Primers used for PCR.

Primer name	Sequence
**ssIGHV_L1_F**	AACTGGGTGGTCTTGTTTGC
**ssIGHG-R**	GGAGTAGAGCCCTGACGG
**IGKV1-F**	GCCTCYTGCTGCTCTGG
**IGKV2-F**	TTCCCTGCTCAGCTCCTG
**IGKC-R**	GACCACCCCATCCACTTTC
**IGLV2-F**	CCTTGTCACCCTCCTCACTC
**IGLV3-F**	CTGGAYCCCTCTCCTGCTC
**IGLV8-F**	CCTGGACGGTGCTTCTGAT
**ssCL4-R**	GCGTCACTGTCTTCTCCACA
**ssIgG156n_1-R_OH**	GTCTCGTGGGCTCGGAGATGTGTATAAGAGACAGCAGAGGGTAGACCGATGGGG
**ssIgG3_1-R_OH**	GTCTCGTGGGCTCGGAGATGTGTATAAGAGACAGGTAGACCGATGGAGCTGTGTTGT
**IGKV1-F_OH**	TCGTCGGCAGCGTCAGATGTGTATAAGAGACAGGCCTCYTGCTGCTCTGG
**IGKV2-F_OH**	TCGTCGGCAGCGTCAGATGTGTATAAGAGACAGTTCCCTGCTCAGCTCCTG
**ssIGKC2_R_OH**	GTCTCGTGGGCTCGGAGATGTGTATAAGAGACAGGGGTCTCTAACTGCTCCTTCG
**ssCL1_R_OH**	GTCTCGTGGGCTCGGAGATGTGTATAAGAGACAGGYGGCCTTGGGCTGAC
**IGLV2-F_OH**	TCGTCGGCAGCGTCAGATGTGTATAAGAGACAGCCTTGTCACCCTCCTCACTC
**IGLV3-F_OH**	TCGTCGGCAGCGTCAGATGTGTATAAGAGACAGCTGGAYCCCTCTCCTGCTC
**IGLV8-F_OH**	TCGTCGGCAGCGTCAGATGTGTATAAGAGACAGCCTGGACGGTGCTTCTGAT
**ssIGA_GSP2**	CTCCAGGTTACTGTCAGGGG
**ssIGHV-F-OH**	TCGTCGGCAGCGTCAGATGTGTATAAGAGACAGGAGGWGAAGCTGGTGGAGTCTG
**ssIGHG-R_OH**	GTCTCGTGGGCTCGGAGATGTGTATAAGAGACAGGGAGTAGAGCCCTGACGG
**ssIgA_GSP2_OH**	GTCTCGTGGGCTCGGAGATGTGTATAAGAGACAGTGGGAAGATTTTGGGGCTGG

**Table 2:** Master mixes for first PCR in nested RT-PCR.

Reagent	IgG (μl)	IgA (μl)	Igκ (μl)	Igλ (μl)
**Nuclease-free water**	9.82	9.82	9.57	9.32
**5X Q5 reaction buffer**	5	5	5	5
**5X Q5 GC enhancer**	5	5	5	5
**10 mM dNTP**	0.5	0.5	0.5	0.5
**10μM ssIGHV_L1_F**	0.25	0.25	—	—
**10μM ssIGHG-R**	0.25	—	—	—
**10μM ssIGA_GSP2**	—	0.25	—	−-
**10μM IGKV1-F**	—	—	0.25	—
**10μM IGKV2-F**	—	—	0.25	—
**10μM IGKC-R**	—	—	0.25	—
**10μM IGLV2-F**	—	—	—	0.25
**10μM IGLV3-F**	—	—	—	0.25
**10μM IGLV8-F**	—	—	—	0.25
**10μM ssCL4-R**	—	—	—	0.25
**Q5 Hot Start High Fidelity DNA polymerase**	0.18	0.18	0.18	0.18
**Template**	4	4	4	4
**Total**	25	24	25	25

**Table 3:** Thermocycler program for first PCR in nested RT-PCR.

	Temperature, °C	Time
**Initial denaturation**	98	30 s
**13 cycles (antibody repertoire)** **30 cycles (single cells)**	98	10 s
	64	20 s
	72	20 s
**Final extension**	72	2 min
**Hold**	4	∞

**Table 4:** Master mixes for second PCR in nested RT-PCR.

Reagent	IgG (μl)	IgA (μl)	Igκ (μl)	Igλ (μl)
**Nuclease-free water**	12.57	12.82	12.57	12.32
**5X Q5 reaction buffer**	5	5	5	5
**5X Q5 GC enhancer**	5	5	5	5
**10 mM dNTP**	0.5	0.5	0.5	0.5
**10μM ssIGHV-F-OH**	0.25	0.25	—	—
**10μM ssIgG156n_1-R_OH**	0.25	—	—	—
**10μM ssIgG3_1-R_OH**	0.25	—	—	—
**10μM ssIGA_GSP2_OH**	—	0.25	—	—
**10μM IGKV1-F_OH**	—	—	0.25	—
**10μM IGKV2-F_OH**	—	—	0.25	—
**10μM ssIGKC2_R_OH**	—	—	0.25	—
**10μM ssCL1_R_OH**	—	—	—	0.25
**10μM IGLV2-F_OH**	—	—	—	0.25
**10μM IGLV3-F_OH**	—	—	—	0.25
**10μM IGLV8-F_OH**	—	—	—	0.25
**Q5 Hot Start High Fidelity DNA polymerase**	0.18	0.18	0.18	0.18
**PCR 1 product**	1	1	1	1
**Total**	25	25	25	25

**Table 5:** Thermocycler programme for second PCR.

	Temperature, °C	Time
**Initial denaturation**	98	30 s
**5 cycles**	98	10 s
	65	20 s
	72	15 s
**25 cycles**	98	10s
	68	20 s
	72	15 s
**Extension**	72	2 m
**Hold**	4	∞

### Sequencing

Samples were sequenced as described previously [17, 42]. Briefly, samples were analysed using a TapeStation 4200 system and D1000 DNA ScreenTape (Agilent Technologies Inc). Samples were pooled and quantified by NEBNext Library Quantification Kit for Illumina (New England Biolabs) and Qubit fluorometer (Thermo Fisher Scientific). Libraries were sequenced on an Illumina NextSeq 2000, with a 40% PhiX control incorporated to increase sequence diversity. Initial quality control was performed using FastQC v0.12.1, then filtering and adapter trimming performed using Trim Galore! v0.6.10 as described previously. Paired-end reads were merged using FLASH v1.2.1. Sequences were annotated for gene usage, isotype and framework using IgBLASTn and IgMAT [43]. Sequences were clustered based on the entire variable region with a 94% identity threshold using UCLUST. Clustering was performed separately for each infection condition, with samples from the uninfected animals included in both analyses. Reads were then normalized to account for animal, time point and tissue sequencing depth variation. Cluster abundances were normalized over the total number of sequences per sample and expressed as percentage of reads.

### Analysis of antibody repertoire

Clusters were partitioned into irrelevant (those unlikely to be antigen-specific) and selected (those which may be antigen-specific) based on kinetics profiles [29]. Due to uncertainty in partitioning, clusters containing less than 10 reads were excluded from analysis.

In the pH1N1 and PRCV Blg20 infection repertoires, clusters containing at least 10% of reads from samples obtained prior to infection or from uninfected animals were defined as irrelevant. Clusters containing less than 10% of reads from samples obtained prior to infection or uninfected animals were defined as selected. This threshold was selected based on previous literature, in which a threshold of 10% facilitated accurate partitioning of clusters with complex kinetics [36].

In the PRCV immunization repertoire, selected clusters were defined as those for which less than 10% of reads originated from samples collected prior to prime immunization with a peak in abundance between 7 and 21 days post-prime immunization, and a second peak 29 days post-prime immunization (4 days post-boost immunization). Peak time points were selected based on previously described kinetics [29].

### Analysis of cluster size and positional diversity

Diversity in cluster size and centroid amino acid sequence was quantified by Shannon and Simpson diversity, two approaches widely used in immune repertoire analysis to capture evenness and richness of clonotypes and sequence variability [44]. Diversity was estimated after randomly subsampling sequences to a common depth across groups to control for sampling bias.

In the pH1N1 and PRCV Blg20 repertoires, indices were calculated for selected and irrelevant clusters containing sequences from single tissues only (PBMC, BAL, TBLN, or spleen) or containing sequences from two or more tissues (shared). For comparison to the immunization repertoire, diversity indices were also calculated for all selected and irrelevant clusters containing sequences from PBMC in the PRCV Blg20 repertoire. In the immunization repertoire, diversity indices were calculated for selected and irrelevant clusters.

Shannon entropy was calculated as $H=-\sum \;p_{i}\log\;p_{i}$, where $p_{i}=\frac{n_{i}}{N}$ represents the proportion of sequences (cluster size) or amino acid frequency (positional diversity) in cluster *i* [45]. Simpson diversity was computed as $D=1-\sum \;p_{i}^{2}$, reflecting the probability that two randomly selected sequences belong to two different clusters [46]. Sequence alignment for positional diversity was performed using Clustal Omega [47]. Shannon index was calculated for each amino acid position in the VDJ sequence (Supplementary Figure S5), then the mean for the entire VDJ sequence calculated.

### Statistical analysis

Tissue distribution, isotype composition, and publicity of clusters was compared between selected and irrelevant clusters using Chi-square test with Yates’ continuity correction. Where expected values were less than five, Fisher exact test was used. All statistical analysis was performed in R v4.5.2.

## Supplementary Material

kyag020_Supplementary_Data

## Data Availability

Data presented in this study are available as BioProject accession numbers PRJNA1471570, PRJNA1471572, and PRJNA1471577.

## Permission to reproduce

Graphical abstract created in BioRender. Briggs, E. (2026) https://BioRender.com/rftqq2o

Supplementary Figure S1 created in BioRender. Briggs, E. (2026) https://BioRender.com/slq3cku

Supplementary Figure S2 created in BioRender. Briggs, E. (2026) https://BioRender.com/yg1sh1h

## Abbreviations

BAL: bronchoalveolar lavage
CoV: coronavirus
HAI: haemagglutination inhibition
IAV: influenza A virus
IIV: inactivated influenza vaccines
LAIV: live attenuated influenza vaccines
N: nucleocapsid protein
PBMC: peripheral blood mononuclear cells
PCV2: porcinecircovirus 2
PEDV: porcine epidemic diarrhoea virus
PFU: plaque formingunits
pH1N1: pandemic H1N1
PRCV: porcine respiratory coronavirus
PRRSV: porcine reproductive and respiratory syndrome virus
RBD: receptor bindingdomain
S: spike protein
SARS-CoV: severe acute respiratory syndromecoronavirus
ST: swine testis
TBLN: tracheobronchial lymph node
