# Supplementary material for: Infection with pandemic H1N1 influenza or porcine respiratory coronavirus drives localized and individualized antibody repertoires, whereas immunization promotes repertoire convergence in pigs
Source: Discov Immunol. 2026 Oct 26;5(1):kyag020. doi: 10.1093/discim/kyag020 (PMC13761479; doi:10.1093/discim/kyag020)
Supplement: Supplementary file 1 [file kyag020_supplementary_data.zip › 2026-09-18_Supplementary Tables.docx]

**Supplementary Table S1. Percentage contributions to Chi-square statistic for tissue distribution in the pH1N1 repertoire.**

| Cluster Type | BAL | PBMC | Shared | Spleen | TBLN |
| --- | --- | --- | --- | --- | --- |
| Selected | 10.21823943 | 23.40411718 | 0.02498746 | 7.72827656 | 4.12713279 |
| Irrelevant | 12.23807071 | 28.03039045 | 0.02992672 | 9.25591885 | 4.94293985 |

**Supplementary Table S2. Percentage contributions to Chi-square statistic for publicity of clusters shared between tissues in the pH1N1 repertoire.**

| Cluster Type | 1 animal | 2 animals | 3-7 animals | 8-14 animals |
| --- | --- | --- | --- | --- |
| Selected | 12.8535928 | 0.4315451 | 10.1269947 | 21.3874974 |
| Irrelevant | 15.8376995 | 0.5317332 | 12.4780909 | 26.3528465 |

**Supplementary Table S3. Percentage contributions to Chi-square statistic for publicity of clusters restricted to a single tissue in the pH1N1 repertoire**

| Cluster Type | 1 animal | 2 animals | 3-7 animals | 8-14 animals |
| --- | --- | --- | --- | --- |
| Selected | 5.035393 | 31.759351 | 4.243491 | 4.713868 |
| Irrelevant | 5.970424 | 37.656805 | 5.031473 | 5.589196 |

**Supplementary Table S4. Percentage contributions to Chi-square statistic for tissue distribution in the PRCV Blg20 repertoire.**

| Cluster Type | BAL | PBMC | Shared | Spleen | TBLN |
| --- | --- | --- | --- | --- | --- |
| Selected | 16.79946192 | 20.86355229 | 0.08499014 | 8.57471383 | 0.27475248 |
| Irrelevant | 19.25284238 | 23.91044937 | 0.09740204 | 9.82695842 | 0.31487713 |

**Supplementary Table S5. Percentage contributions to Chi-square statistic for publicity of clusters shared between tissues in the PRCV Blg20 repertoire.**

| Cluster Type | 1 animal | 2 animals | 3-7 animals | 8-14 animals |
| --- | --- | --- | --- | --- |
| Selected | 7.560177 | 3.158265 | 11.231806 | 23.380248 |
| Irrelevant | 9.117728 | 3.808932 | 13.545787 | 28.197057 |

**Supplementary Table S6. Percentage contributions to Chi-square statistic for publicity of clusters restricted to a single tissue in the PRCV Blg20 repertoire.**

| Cluster Type | 1 animal | 2 animals | 3-7 animals | 8-14 animals |
| --- | --- | --- | --- | --- |
| Selected | 2.902238 | 28.832527 | 13.791626 | 1.473058 |
| Irrelevant | 3.272809 | 32.513994 | 15.552603 | 1.661145 |

**Supplementary Table S7. Percentage contributions to Chi-square statistic for isotype composition of clusters in the PRCV 135 immunisation repertoire.**

| Cluster Type | 90-100% IgG | 80-90% IgG | 70-80% IgG | 60-70% IgG | 50-60% IgG | 40-50% IgG | 30-40% IgG | 20-30% IgG | 10-20% IgG | 0-10% IgG |
| --- | --- | --- | --- | --- | --- | --- | --- | --- | --- | --- |
| Selected | 2.44 | 39.70 | 3.95 | 1.47 | 2.29 | 1.09 | 5.33 | 7.76 | 31.69 | 3.14 |
| Irrelevant | 0.03 | 0.46 | 0.05 | 0.02 | 0.03 | 0.01 | 0.06 | 0.09 | 0.37 | 0.04 |

**Supplementary Table S8. Adjusted p values for Fisher Exact tests for differences in distribution in publicity in the PRCV 135 immunisation repertoire.**

| Number of animals | 1 animal | 2 animals | 3 animals | 4 animals | 5 animals |
| --- | --- | --- | --- | --- | --- |
| P value | 1.729634e-08 | 1.175503e-06 | 0.05694419 | 0.1438030 | 0.05694419 |

**Supplementary Table S9. Original and updated nomenclature for porcine IGHV gene segments.**

| Original nomenclature | IMGT nomenclature |
| --- | --- |
| VHG | IGHV1-2 |
| VHA | IGHV1-4 |
|  | IGHV1-5 |
| VHB | IGHV1-6 |
| VHE | IGHV1-8 |
| VHA* | IGHV1-10 |
| VHF | IGHV1-11 |
| VHB* | IGHV1-12 |
| VHY | IGHV1-14 |
| VHN | IGHV1-15 |
| VHC | IGHV1S2 |
| VHI | IGHV1S3 |
| VHE | IGHV1S5 |
| VHF | IGHV1S6 |
| VHK | IGHV1S7 |
| VHH | IGHV1S8 |
